# Charge Transport in Carbon Nanotubes-Polymer Composite Photovoltaic Cells

**DOI:** 10.3390/ma2030710

**Published:** 2009-06-29

**Authors:** Adnen Ltaief, Abdelaziz Bouazizi, Joel Davenas

**Affiliations:** 1 Laboratoire de Physique et Chimie des Interfaces, Faculté des Sciences de Monastir, Avenue de l’Environnement, 5019 Monastir, Tunisia; E-Mail: bouazizi@fsm.rnu.tn (A.B.); 2 Ingénierie des Matériaux Polymères, CNRS - Université Claude Bernard-Lyon 1, 43 Bd du 11 Novembre, 69100 Villeurbanne, France; E-Mail: joel.davenas@uni-lyon1.fr (J.D.)

**Keywords:** carbon nanotubes, MEH-PPV, conduction mechanisms, photovoltaic response

## Abstract

We investigate the dark and illuminated current density-voltage (J/V) characteristics of poly(2-methoxy-5-(2^’^-ethylhexyloxy)1-4-phenylenevinylene) (MEH-PPV)/single-walled carbon nanotubes (SWNTs) composite photovoltaic cells. Using an exponential band tail model, the conduction mechanism has been analysed for polymer only devices and composite devices, in terms of space charge limited current (SCLC) conduction mechanism, where we determine the power parameters and the threshold voltages. Elaborated devices for MEH-PPV:SWNTs (1:1) composites showed a photoresponse with an open-circuit voltage V_oc_ of 0.4 V, a short-circuit current density J_SC_ of 1 µA/cm² and a fill factor FF of 43%. We have modelised the organic photovoltaic devices with an equivalent circuit, where we calculated the series and shunt resistances.

## 1. Introduction 

Solar cells based on conjugated polymers [1,2] are attractive due to their potential for low-cost manufacture. Power conversion efficiencies are currently approaching the values required for commercial viability, with a considerable amount of research being carried out to further improve the efficiencies. One of the main areas highlighted for improvement is the role of charge generation and transport in such devices.

The mechanism of charge generation in organic solar cells differs from that in conventional inorganic devices. In the former, light absorption results in the production of excitons, as opposed to the free electron-hole pairs which are formed in the latter [3,4]. For more efficiencies of the organic photovoltaic (OPV) response, the photogenerated excitons must dissociate at the interfaces between electrons conducting and holes conducting materials. The typical diffusion length of excitons, however, is only around 20 nm [5], and thus important to maximise the interface area for a given device so as to increase the chances of excitons encountering the interface. This fact, has investigated the development of bulk heterojunction organic solar cells containing blends of electrons and holes conducting materials, in which the fine detail of the structure creates the large interface area required [6].

Experiments performed on these so called bulk heterojunction devices have shown that the large interface area created does indeed lead to efficient excitons dissociation, with evidence to suggest that complex composite morphologies can effect charge dissociation efficiencies [7,8]. Despite this, the overall power conversion efficiencies, while much improvements, are not as high as would be expected. This means that poor transport in the device is allowing the photo-generated charge to recombine before reaching the respective electrodes. Polymers have been used previously in photovoltaic cells, but the low electrons mobilities of most conjugated polymers only allows them for use as the hole conducting components in a blend with other materials, such as fullerene [7], organic dyes [9] or in the present work with carbon nanotubes (CNTs). In these composite materials, the photocurrent is typically increased by several orders of magnitudes with comparaison to polymer only devices.

In order to develop and improve device architectures that can be incorporated into useful electronic devices, it is necessary to understand (i) the fundamental physical properties of the carbon nanotubes (CNTs) in composites with conjugated polymer and (ii) the operating principales of such alternative devices. At the nanoscale, many quantum mechanical processes become important and the application of these properties can lead to understand behaviours that are not observed in bulk devices.

The transport properties in thin device structures made of conjugated polymers have been well characterized using space charge limitted current models with field dependent mobilities [10]. The carrier transport behaviour of MEH-PPV and single-walled carbon nanotubes (SWNTs) materials in composite devices, in particular photovoltaic cells, has not been sufficiently characterized. In this work, we try to give a better insight into the conduction mechanism in MEH-PPV:SWNT photovoltaic composite by fabricating and analysing bulk heterojunction polymer-SWNTs photovoltaic cells, the poly(2-methoxy-5-(2^’^-ethylhexyloxy)1-4-phenylenevinylene) (MEH-PPV) as the electron donor material. The dark and illuminated current density-voltage (J/V) of the devices are examined to identify the role of SWNTs doping in carrier transport. The observed space-charge limited current (SCLC) of the photovoltaic cells is explained based on an effective exponential tail state for the polymer matrix, which is modified by the embedded SWNTs.

## 2. Results and Discussion

**Figure 1:**
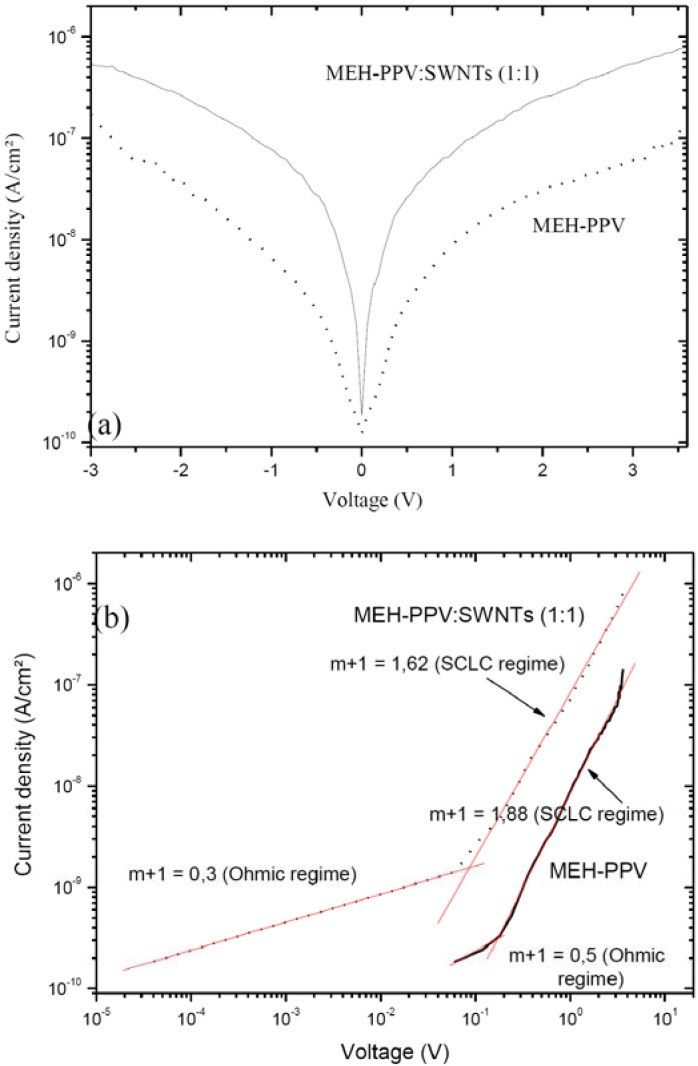
(a) Dark J-V characteristics of ITO/MEH-PPV/Al and ITO/MEH-PPV:SWNTs (1:1)/Al devices plotted on a semi-logarithmic scale, (b) same characteristics in logarithmic scale.

Figure 1(a) shows in a semi-logarithmic plot the dark current density –voltage (J-V) characteristics of ITO/MEH-PPV/Al and of ITO/MEH-PPV :SWNTs (1:1)/Al devices. For positive voltages (V_D_ > 0), the dark current increases exponentially in a subthreshold regime followed by a power-law regime at higher voltages. The power-law current-voltage characteristics are attributed to a space-charge limited current (SCLC) of the carriers in both polymer and composite materials. 

As seen from Figure 1(a), for positive voltages, the dark current of the sample doped with SWNT is close to two orders of magnitude higher than that of the only polymer sample, due to the improved carrier conduction and mobility. The reverse current of the device with SWNTs is also higher to two orders of magnitude than that of the MEH-PPV only device. To shed light on the mechanisms responsible for the improved dark conductivity of the polymer doped with SWNTs, we first present the hopping transport mechanism in the MEH-PPV only device. Then, by comparaison of the electrical characteristics of the MEH-PPV-SWNTs and MEH-PPV only devices, we investigate the effect of SWNTs doping on the electronic properties of the polymer matrix.

The dark current density-voltage J/V characteristics in logarithmic scale are presented in Figure 1 (b). Analysis of these curves in general reveals that various conduction regimes can be discriminated, following the power-law dependence J ~ V^m+1^ [4], namely the space charge limited current (SCLC) model. SCLC theory predicts the crossover from the ohmic regime (m = 0) at low voltages to the SCLC regime governed by shallow-trap (m = 1) and to trap-filled regime (m >> 1). For our case, it can be seen in Figure 1(b) that the ohmic regime governs conduction mechanism at low bias voltages with low values of (m+1) exponent, for both structures (only MEH-PPV and MEH-PPV:SWNTs composite). This is probably due to a low intrinsic conductivity of our polymer. For higher applied voltages, SCLC regime appears to control conduction mechanism. 

### 2.1. Hopping conduction in only MEH-PPV device

Hopping of carriers between localized electronic states of the MEH-PPV polymer constitute the main mechanism for carrier transport. These localized states trap most of the carriers and are present due to the disorder in the electronic overlap and linkage of MEH-PPV polymer; which is related also to the morphology induced by the used solvents [11]. The density of localized states in the mobility gap of a polymer determines the density of trapped carriers and influences the effective conductivity of the material. It has been reported that for limited movement of Fermi energy, the density of localized states can be approximated by an exponential trap density [12,13,14] as following:

(1) $$g\left(E\right)=\;\frac{N_{t}}{kT_{t}}\exp\left(\frac{E}{kT_{t}}\right)$$

Here, E is the energy and it is negative with respect to the hopping transport band edge [14], N_t_ is the total density of traps, T_t_ is the characteristic slope of the exponential tail states and k the Boltzmann’s constant. In a diode consisting of injecting electrodes that sandwich the organic semiconductor material, the SCLC (Space Charge Limited Current) current density due to the carriers excited to the hopping transport band can be written as:

(2) $$J=q\mu_{b}N_{b}\tau {\left(\frac{\varepsilon }{qN_{t}}\right)}^{\alpha }\frac{{\left(V_{D}-V_{t}\right)}^{\alpha +1}}{t^{2\alpha +1}}$$

where q is the elementary charge, N_b_ the density of states in the hopping transport band, μ_b_ the carrier mobility of the hopping band, ε the dielectric constant, V_D_ the diode voltage, t thickness of the organic material, α = T_t_/T the power parameter, T the temperature, V_t_ the threshold voltage and τ = α^α^(2α+1)^α+1^/(α+1)^2α+1^ [15,16]. Equation (2) is derived from Poisson’s and charge conservation equations considering both mobile and trapped carriers. As seen in (2), the current-voltage characteristics follow a power-law dependence, with a power parameter that is a function of the characteristic slope of the tail states T_t_ normalized by the ambient temperature.

As a result, there exists a clear link between the exponentiel distribution of the localized states and the observed power-law behaviour of the only MEH-PPV device. Although, the electrical characteristics are often influenced by non-ideal parameters such as contact resistance. They provide approximate informations on the electronic properties of the used material; including density of states and effective mobility.

For the determination of the power parameter α from the current-voltage characteristics, we use a method described in [18]. Power-law J-V characteristics have a general form:

(3) $$J=k{\left(V_{D}-V_{t}\right)}^{\alpha +1}$$

where Vt is the onset of the power-law regime and k the current prefactor. To extract α, the slope of the J/g versus V plot can be used since $\frac{J}{g}=\frac{V-V_{t}}{\alpha +1}$, where g indicates the conductance defined by $g=\;\frac{\partial J}{\partial V}$ [17].

### 2.2. Comparison of MEH-PPV:SWNTs and MEH-PPV devices

**Figure 2:**
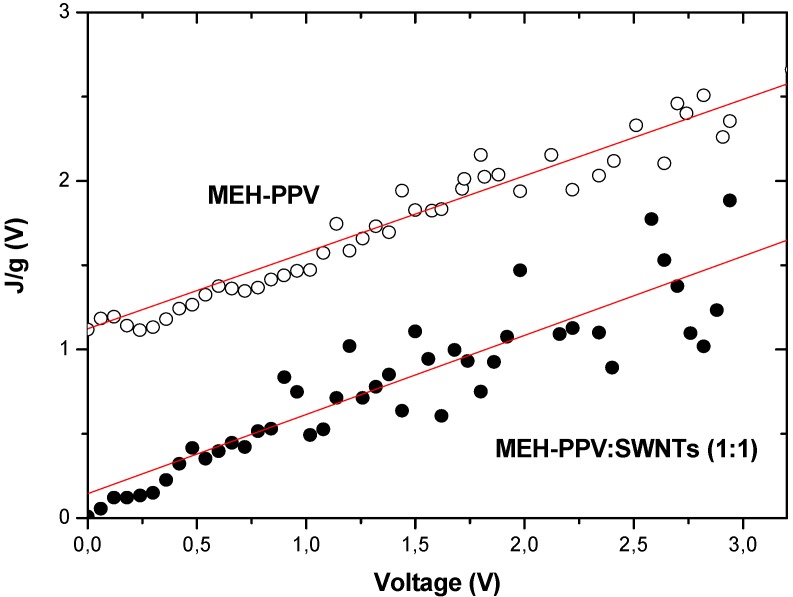
J/g of MEH-PPV and MEH-PPV+SWNTs devices shown in Figure 1 as a function of diode voltage used for the extraction of power parameters and threshold voltage of the SCLC regime. The MEH-PPV curve is shifted by +1 for better readability. The lines are used to determine the slopes.

Considering nanotubes as a dopant in the MEH-PPV polymer matrix due to their relatively low density, we use a similar exponential band tail model for the polymer-nanotubes composite devices. This provides an effective representation of the influence of single-walled carbon nanotubes doping on the electronic properties of the polymer matrix. Figure 2 illustrates the J/g versus V_D_ curve for the forward portion of the J-V characteristics of MEH-PPV-SWNTs and MEH-PPV devices shown in Figure 1. As seen from Figure 2, there is a significant increase in the J/g values of MEH-PPV only devices in comparison to those of composite devices. Power parameter of 1.125 and T_t_ = 337 K for MEH-PPV:SWNTs (1:1) composite devices, have been calculated. Due to the presence of variance in the slope of the curve, a maximum error of 10 % is estimated in the value of α and T_t_. The extracted parameters for this power-law regime are summarized in table 1. The characteristic temperature of T_t_ = 337 K indicates a low density of states in the middle of the gap. 

For the MEH-PPV diode, a high power parameter of 1.25 can be extracted for a wide range of positive voltages. This indicates that the density of tail states decays at a slow rate corresponding to T_t_ = 375 K. In summary, due to a slower increase in current, we expect a higher density of localized states in the bandgap of the MEH-PPV polymer device as compared to the MEH-PPV-SWNTs device. Power-law parameters in SCLC regime for P3OT and P3OT :SWNTs (1%) composite devices are 1.981 and 1.056 for the power parameter and 595 and 317 for the characteristic temperature [18].

The obtained results show that the band tail in composite device decreases, which correlates with the lower characteristic temperature and the higher current level. Despite their limited accuracy, the above results indicate that incoporation of SWNTs in MEH-PPV polymer matrix contributes to a reduction of the localized states in the bandgap of the polymer, resulting in improved carrier conduction and mobility.

**Table 1:** Extracted parameters for the dark SCLC regime of MEH-PPV and MEH-PPV:SWNTs (1:1) devices.

Parameter	MEH-PPV	MEH-PPV:SWNTs (1:1)
α	1.250	1.125
T_t_ (K)	375	337
V_t_ (V)	0.26	1.21

### 3.3. Characterization of photovoltaic response in MEH-PPV:SWNTs composite

**Figure 3:**
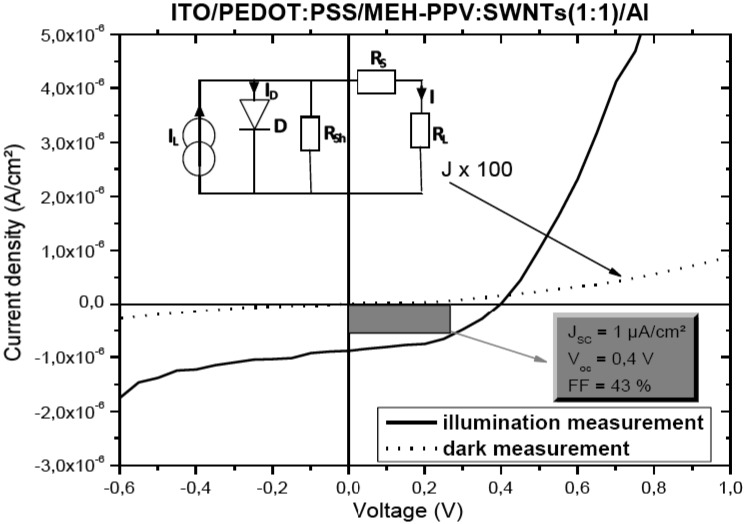
Current density-voltage characteristics of ITO/PEDOT:PSS/MEH-PPV:SWNTs(1:1)/Al cells in the dark (x 100 for more readability) and under illumination. In the inset, is a schematic view of the equivalent circuit for a typical photovoltaic cell.

Figure 3 shows current density-voltage characteristics of ITO/PEDOT:PSS/MEH-PPV:SWNTs (1:1)/Al devices in the dark and under illumination. The photovoltaic parameters, such as the short circuit current density (J_SC_), the open circuit voltage (V_OC_) and fill factor (FF) can be extracted from the characteristic J-V plots. The J_SC_, V_OC_ and FF of the MEH-PPV:SWNTs (1:1) sample when exposed to light are, respectively, about 1 µA/cm², 0.4 V and 43 %. These findings propose that the photovoltaic response of the blend device is due to the introduction of internal polymer/nanotubes junctions act as dissociation centers, which are able to split up the excitons and also create a continuous pathway for the charge carriers to be efficiently transported to the corresponding electrodes.

Thin film photovoltaic cells can be modelled as a simple photoactive diode, with an equivalent circuit as shown in the inset of Figure 3. Where, R_s_ is the series resistance, R_sh_ is the shunt resistance; arising from pinholes and other short circuit pathways; R_L_ is the load resistance for external circuit and I_L_ is the photocurrent. The major loss of organic photovoltaic power through lowered J_SC_ values originates from high values of R_s_. This series resistance is expected to rise from (i) the linear addition of contact resistances and the resistance to charge flow within the TCO film itself, (ii) low charge mobilities within the thin organic layers and (iii) low rates of charge transfer between donor and acceptor materials and charge transport to corresponding electrodes [19]. The shunt resistance is defined to be the reverse slope of the J/V curve at the 0 V point:

(4) $$R_{sh}=\lim_{V\to 0}\frac{dV}{dJ}$$

Knowing the value of the shunt resistance is important because of the nature of electronic current to flow in the path of least resistance. For our elaborated devices we find a R_sh_ value of about 0.7 MΩ cm². The series resistance is defined to be the reverse slope of the J/V curve at the 0 A/cm² point:

(5) $$R_{s}=\lim_{I\to 0}\frac{dV}{dJ}$$

In our case, we calculate a R_s_ value of about 80 kΩ cm². The relatively high values of series and shunt resistances determined for MEH-PPV:SWNTs composite photoactive layer, could be attribute to the formation of shallow hole traps in the polymer matrix. Also, the presence of metallic SWNTs accentuates this increase in R_s_ and R_sh_ resistances, as they have no bandgap and can act as recombination and trap centers in the gap of the composite semiconductor medium, which block hole transport in the MEH-PPV polymer matrix.

## 3. Experimental Section 

The MEH-PPV conjugated polymer and the SWNTs (Carbolex AP-grade, 12-15 angstrom diameter) were purchased from Aldrich. MEH-PPV has a molar mass of 86 000 g/mol determined by gel permeation chromatography (GPC). The ITO substrates were cleaned in an ultrasonic bath of acetone (HPLC grade) for 20 min, followed by isopropyl alcohol (IPA, HPLC grade) rinsing for 20 min at room temperature, before being dried in an nitrogen gas flow.

MEH-PPV:SWNTs (1:1) composite solutions were prepared by blending the MEH-PPV with the SWNTs in an appropriate solvent, at a concentration of 15 mg/mL, and dispersing the SWNTs by using a magnetic stirrer. Sonication for 30 min is sufficient to give a stable transparent solution. 

In the first part of this work, the organic active layer of MEH-PPV or MEH-PPV:SWNTs (1:1) composite, is sandwiched between two electrodes on a glass substrate. The bottom electrode is an ITO layer, which serves as the anode and the top electrode is an Al layer, which is used as the cathode. In forword bias condition, the positive voltage was applied to the ITO layer with respect to the metal electrode.

Photovoltaic cells were fabricated by spin coating (2,000 rpm for 20 s) the active bulk heterojunction layer onto a 100 nm precleaned ITO glasses of 1 cm × 1 cm coated with PEDFOT:PSS layer, using a “P6700 Series” spin coater. A layer of PEDOT:PSS between the transparent ITO electrode and the photoactive layer has been deposited from an aqueous solution onto the ITO substrate with an angular speed of 1,500 rpm. PEDOT:PSS films were dried on a hot plate under a nitrogen atmosphere for 10 min at 120 °C.

Completion of the photovoltaic device occurs when aluminium contacts (typical thickness ~ 170 Å) are applied to the MEH-PPV:SWNTs composite film layers. This is accomplished using thermal evaporation under vacuum at pressures <10^-6^ mbar with a standard shadow mask. The approximately thickness of the polymer/SWNTs layers was of the order of 500 nm. The effective solar cell area as defined by the geometrical overlap between the bottom ITO electrode and the top cathode was 0.1 cm^2^.

The short circuit current and I-V curves were measured using a “Keithley 6430 SUB-FEMTOAMP REMOTE Sourcemeter” source unit, using an Halogen lamp for illumination. Photovoltaic cell testing was performed in an isolated black box configuration to assure standard calibration and reproducibility of results. All processing steps were carried out in clean room conditions. 

## 4. Conclusions 

We have investigated the effect of SWNTs doping on the charge transport mechanisms of MEH-PPV and MEH-PPV:SWNT (1:1) devices. Hopping of carriers between localized electronic states of the MEH-PPV polymer constitute the main mechanism for carrier transport. A clear link between the exponentiel distribution of the localized states and the observed power-law behaviour of the only MEH-PPV device, has been identified. The observed space-charge limited current (SCLC) regime of the photovoltaic cells is explained by an exponential tail state model for the polymer matrix, which is modified by the embedded SWNTs. The ability to construct organic D/A network composite photovoltaic cells containing single-walled carbon nanotubes (SWNTs)-polymer (MEH-PPV) composites has been demonstrated. The relatively high values of series and shunt resistances can be explained by a reduced photogeneration rate and increased recombination rate, which is exacerbated by the proportional metallic nanotubes in the composite layers.
